# The prognostic value of admission serum uric acid for acute kidney injury: a two-center retrospective analysis

**DOI:** 10.3389/fmolb.2025.1635227

**Published:** 2025-06-18

**Authors:** Shuo Huang, Linger Tang, Lingyi Xu, Jinwei Wang, Xizi Zheng

**Affiliations:** ^1^ Renal Division, Peking University First Hospital, Beijing, China; ^2^ Institute of Nephrology, Peking University, Beijing, China; ^3^ Key Laboratory of Renal Disease, Ministry of Health of China, Beijing, China; ^4^ Key Laboratory of CKD Prevention and Treatment, Ministry of Education of China, Beijing, China; ^5^ Research Units of Diagnosis and Treatment of Immune-Mediated Kidney Diseases, Chinese Academy of Medical Sciences, Beijing, China

**Keywords:** acute kidney injury, serum uric acid, hyperuricemia, hypouricemia, estimated glomerular filtration rate

## Abstract

**Background:**

Acute kidney injury (AKI) is a serious clinical syndrome, with elevated serum uric acid (SUA) recognized as a potential modifiable risk factor. Nonetheless, the association between reduced SUA and the risk of AKI, along with the modification by kidney function on this association, is not well understood.

**Methods:**

All adult patients from Peking University First Hospital (PKUFH) were screened. The primary outcome was AKI during hospitalization. Restricted cubic spline (RCS) was utilized to examine the hypothesized non-linearity between AKI and SUA as a continuous variable. SUA was categorized into six groups and Poisson regression was applied to evaluate the association between SUA groups and AKI with 240–360 μmol/L as reference. Subgroup analysis was conducted in terms of estimated glomerular filtration rate (eGFR).

**Results:**

Among 62,775 patients enrolled from PKUFH, 1,866 patients developed AKI (3.0%). The RCS plot showed a U-shaped association between SUA and AKI. Compared with reference group, SUA ≤ 180 μmol/L and >480 μmol/L exhibited a 2.17-fold and a 4.86-fold increased risk of AKI in the unadjusted model. After full adjustment, the associated risk of AKI in SUA ≤ 180 μmol/L (RR 1.92, 95% CI 1.57–2.36) and SUA > 480 μmol/L (RR 1.17, 95% CI 1.03–1.34) was weakened but still demonstrated statistical significance. When stratified by eGFR, the U-shaped risk curve was much less steep in the subgroup with eGFR ≤ 45 mL/min/1.73 m^2^.

**Conclusion:**

This study reveals a U-shaped association between admission SUA and AKI risk. Kidney function is an important confounder for this association.

## 1 Introduction

Acute kidney injury (AKI) constitutes a critical clinical syndrome affecting more than 20% of hospitalized patients worldwide (Susantitaphong et al., 2013), leading to considerable morbidity and mortality. Currently, there are no established therapeutic interventions for AKI, highlighting the urgent need to identify modifiable risk factors to improve prognosis. Elevated serum uric acid (SUA) is recognized as one such risk factor. Aside from crystal precipitation and obstruction in tubules (Spencer et al., 1976), the biological plausibility of uric acid as a potential toxin affecting the kidneys is supported by numerous *in vitro* and *in vivo* studies demonstrating its capability to induce inflammation (Meotti et al., 2011), oxidative stress (George and Struthers, 2009), activation of the renin-angiotensin system (Zhang et al., 2015), and vascular remodeling (Kirca et al., 2017).

In contrast to the thorough investigation into the association between elevated SUA and AKI across various clinical contexts such as cardiac surgery (Gaipov et al., 2015), acute leukemia (Ejaz et al., 2015) and contrast exposure (Park et al., 2011; Mendi et al., 2017), the impact of reduced SUA on AKI risk remains poorly understood with inconsistent findings reported (Otomo et al., 2016; Cheungpasitporn et al., 2016). The variations in baseline kidney function among the study population may elucidate this inconsistency, as the modified effect of baseline kidney function on the association between SUA and long-term kidney prognosis has been noted (Meier-Kriesche et al., 2009; Srivastava et al., 2018). To conduct a comprehensive investigation into the association between various SUA levels and AKI in hospitalized patients while also accounting for the potential confounding effect of baseline kidney function on this association, we initiated this study using a two-center design with an external validation cohort to corroborate the findings across diverse populations.

## 2 Materials and methods

### 2.1 Datasets

Data were obtained from two independent databases: the Peking University First Hospital (PKUFH) database as the development cohort and the Medical Information Mart for Intensive Care IV database (MIMIC-IV) as the external validation cohort. PKUFH database recorded all 210,550 admissions from 2018 to 2021 in PKUFU, a large academic medical center hospital in Beijing, China. MIMIC-IV contains 180,747 de-identified electronic health records from Beth Israel Deaconess Medical Center from 2008 to 2019. The PKUFH database and the MIMIC-IV database were approved by the Clinical Research Ethics Committee of Peking University First Hospital (2025 063-001) and the institutional review boards of the Beth Israel Deaconess Medical Center (2001-P-001699/14) and the Massachusetts Institute of Technology (No. 0403000206).

### 2.2 Study population

All patients with at least one measurement of SUA within 48 h of admission and two measurements of serum creatinine (SCr) during hospitalization were included in this study. Exclusion criteria were as follows (Susantitaphong et al., 2013): age less than 18 years (Spencer et al., 1976), long-term dialysis or an estimated glomerular filtration rate (eGFR) less than 15 mL/min/1.73 m^2^ at admission (Meotti et al., 2011), kidney resection or transplantation (George and Struthers, 2009), AKI developed within 48 h of admission, and (Zhang et al., 2015) hospital stay shorter than 48 h. AKI was defined based on the KDIGO guidelines as an increase in SCr by 26.5 μmol/L or more within 48 h or an increase of at least 1.5 times the reference SCr within 7 days (Khwaja, 2012).

### 2.3 Data collection

Data from PKUFH were systematically collected through chart abstraction utilizing the institution’s clinical data warehouse, while data from the MIMIC-IV database were retrieved through Structured Query Language. The initial SUA level obtained within 48 h of admission was recorded. Chronic comorbidities were identified based on diagnoses at admission or discharge, in accordance with the International Classification of Diseases, 10th Revision (ICD-10) codes. Cardiovascular diseases include conditions such as coronary artery disease and chronic heart failure. Laboratory indices and prescribed medications were documented if they were measured or administered within 48 h of admission, respectively. ICU admission was defined as the admission or transfer of patients to the ICU within 48 h following their initial admission.

### 2.4 Exposure and outcome

The primary exposure was admission SUA, which was measured within 48 h of admission by standard laboratory procedures using the uricase/peroxidase enzymatic methods (AU5800; Beckman Coulter, United States) and measured at the PKUFH Central Laboratory. The primary outcome is AKI during hospitalization, defined by KDIGO SCr criteria.

### 2.5 Statistical analysis

Categorical variables were presented as frequencies and analyzed using the Chi-square test or Fisher’s exact test. Continuous variables were expressed as mean ± SD or medians (interquartile range) depending on their distribution and compared using non-parametric tests. Multiple imputations were used to address missing values. Linear regression was used to examine the association between SUA and kidney function (SCr and eGFR).

Given that prior research has suggested a potential non-linear association between SUA and AKI, a restricted cubic spline (RCS) analysis was conducted to examine this proposed non-linearity, treating SUA as a continuous variable. The optimal number of knots in the spline was identified by selecting the lowest Akaike Information Criterion (AIC) value. To test for non-linearity, a likelihood ratio test was employed, comparing a model that considered only linear factors to one that included both linear and cubic spline components.

A multivariable Poisson regression analysis was performed to assess the association between SUA groups and AKI. Admission SUA levels were utilized to categorize patients into six groups: ≤180, 180–240, 240–360, 360–420, 420–480 and >480 μmol/L. The normal SUA range of 240–360 μmol/L was defined as the reference group (Richette et al., 2017). This analysis adjusted for age, gender, body mass index (BMI), eGFR at admission, hypertension, diabetes, cardiovascular disease, liver cirrhosis, malignancy, diuretics, nonsteroidal anti-inflammatory drugs (NSAIDs), contrast, SUA-lowering medications, ICU admission, albumin, prealbumin, and hemoglobin. Risk ratios (RRs) with 95% confidence intervals (CIs) were calculated to quantify the associations.

A subgroup analysis was conducted to determine whether differences existed in eGFR subgroups concerning the association between SUA and AKI. An eGFR of 45 mL/min/1.73 m^2^ was chosen as the discrimination value based on evidence indicating that the clinical impact of elevated SUA and SUA-lowering therapies was limited in patients with this level of kidney function loss (≥stage 3b CKD) (Srivastava et al., 2018; Maahs et al., 2013). We performed sensitivity analysis by constraining AKI to a 7-day period within the hospital stay because changes in disease conditions and medications during hospitalization might affect SUA levels. Furthermore, we excluded patients receiving uric acid-lowering medications that could substantially alter SUA levels.

External validation was conducted by reanalyzing the MIMIC-IV database. Patients were categorized into six groups based on admission SUA levels, using the same criteria as in the PKUFH cohort. Subsequently, multivariable Poisson regression was applied, adjusting for the same covariates as in the PKUFH cohort, except for prealbumin, which was not available in the MIMIC-IV database.

## 3 Results

### 3.1 Baseline characteristics of the development cohort

During the period from 1 January 2018, to 31 December 2020, a total of 210,550 patients were admitted to PKUFH. Of these, 62,775 admissions met the criteria for inclusion in this study (Supplementary Figure 1A). The study population had a median age of 61 (48, 70) years old, and 55.5% of the participants (n = 34,818) were male. The most common comorbidities were hypertension (n = 28,854, 46.0%), diabetes (n = 15,767, 25.1%), and cardiovascular disease (n = 15,292, 24.4%). The median SUA was 329 (266, 402) μmol/L, and the median eGFR was 92.1 (74.8, 103.5) mL/min/1.73 m^2^. The linear regression analysis revealed significant associations between serum uric acid and kidney function: a positive relationship with SCr (β = 0.40, 95% CI [0.39, 0.41], *P* < 0.001) and an inverse relationship with eGFR (β = −1.74, 95% CI [−1.77, −1.71], *P* < 0. 001). SUA-lowering medication was prescribed in 2.1% of the entire population. Additional information is available in Table 1.

**TABLE 1:** Baseline Characteristics of PKUFH patients.

Variables	Total (N = 62,775)	≤180 (N = 3,011)	180–240 (N = 7,514)	240–360 (N = 28,344)	360–420 (N = 11,272)	420–480 (N = 6,516)	>480 (N = 6,118)
Age (years)	61 (48, 70)	62 (47, 72)	60 (45, 70)	62 (50, 70)	61 (49, 71)	60 (47, 70)	60 (43, 71)
Male	34,818 (55.5)	1,079 (35.8)	2,528 (33.6)	14,359 (50.7)	7,640 (67.8)	4,797 (73.6)	4,415 (72.2)
BMI (kg/m^2^)	24.5 (22.1, 27.1)	22.2 (19.8, 24.6)	22.9 (20.6, 25.4)	24.3 (22.0, 26.8)	25.1 (22.9, 27.7)	25.6 (23.3, 28.1)	25.7 (23.1, 28.4)
Baseline eGFR (mL/min/1.73 m^2^)	92.1 (74.8, 103.5)	100.7 (91.7, 111.8)	98.9 (88.4, 110.3)	94.3 (81.1, 104.1)	88.2 (70.4, 100.6)	82.3 (61.1, 98.5)	64.5 (38.2, 89.3)
Baseline SCr (μmol/L)	75.1 (62.9, 90.4)	58.4 (50.0, 68.3)	62.7 (55.2, 72.9)	71.5 (61.7, 83.2)	81.4 (70.7, 95.0)	88.1 (75.9, 106.7)	104.6 (84.1, 156.6)
ICU admission	4,785 (7.6)	320 (10.6)	522 (6.9)	1,968 (6.9)	831 (7.4)	521 (8.0)	623 (10.2)
Comorbidities, n (%)							
Hypertension	28,854 (46.0)	983 (32.6)	2,593 (34.5)	12,331 (43.5)	5,614 (49.8)	3,571 (54.8)	3,762 (61.5)
Diabetes	15,767 (25.1)	713 (23.7)	1,775 (23.6)	6,975 (24.6)	2,880 (25.6)	1,667 (25.6)	1,757 (28.7)
Cardiovascular disease	15,292 (24.4)	492 (16.3)	1,327 (17.7)	6,573 (23.2)	2,977 (26.4)	1,841 (28.3)	2,082 (34.0)
Liver cirrhosis	933 (1.5)	72 (2.4)	140 (1.9)	379 (1.3)	137 (1.2)	88 (1.4)	117 (1.9)
Malignancy	3,280 (5.2)	177 (5.9)	414 (5.5)	1,527 (5.4)	600 (5.3)	303 (4.7)	259 (4.2)
Medication, n (%)							
Diuretic	6,889 (11.0)	323 (10.7)	493 (6.6)	2,311 (8.2)	1,274 (11.3)	925 (14.2)	1,563 (25.5)
NSAIDs	11,935 (19.0)	559 (18.6)	1,305 (17.4)	5,413 (19.1)	2,200 (19.5)	1,327 (20.4)	1,131 (18.5)
Contrast	4,040 (6.4)	222 (7.4)	535 (7.1)	1,920 (6.8)	709 (6.3)	382 (5.9)	272 (4.4)
SUA-lowering medication	1,308 (2.1)	36 (1.2)	83 (1.1)	406 (1.4)	217 (1.9)	197 (3.0)	369 (6.0)
Lab testing							
Hb (g/L)	132.0 (117.0, 144.0	116.0 (100.0, 129.0)	125.0 (112.0, 136.0)	132.0 (120.0, 143.0)	136.0 (122.0, 148.0)	137.0 (121.0, 150.0)	131.0 (109.0, 147.0)
Alb (g/L)	40.6 (36.3, 43.9)	35.8 (31.3, 40.4)	39.4 (34.9, 43.0)	40.9 (37.1, 44.0)	41.5 (37.5, 44.4)	41.3 (37.0, 44.4)	39.7 (34.3, 43.7)
Prealbumin (mg/L)	226.4 (176.4, 270.3)	148.3 (92.3, 210.9)	200.9 (147.0, 240.1)	225.0 (180.6, 264.6)	242.0 (195.3, 282.0)	250.5 (200.2, 294.5)	247.9 (182.1, 300.0)
Sodium (mmol/L)	139.7 (137.7, 141.2)	138.0 (135.1, 140.2)	139.1 (136.8, 140.9)	139.8 (137.9, 141.3)	139.9 (138.1, 141.3)	139.8 (138.1, 141.2)	139.4 (137.4, 141.1)
Potassium (mmol/L)	3.8 (3.6, 4.1)	3.7 (3.4, 4.0)	3.8 (3.5, 4.0)	3.8 (3.6, 4.1)	3.9 (3.6, 4.1)	3.9 (3.6, 4.2)	4.0 (3.7, 4.3)
Chloride (mmol/L)	105.2 (103.3, 107.0)	103.9 (100.7, 106.4)	104.9 (102.8, 106.8)	105.3 (103.4, 107.0)	105.4 (103.6, 107.1)	105.4 (103.5, 107.3)	105.3 (102.7, 107.5)
Phosphate (mmol/L)	1.1 (1.0, 1.2)	1.0 (0.8, 1.1)	1.1 (0.9, 1.2)	1.1 (1.0, 1.2)	1.1 (1.0, 1.2)	1.1 (1.0, 1.2)	1.1 (1.0, 1.3)

BMI, body mass index; eGFR, estimated glomerular filtration rate; sCr, serum creatinine; ICU, intensive care unit; NSAIDs, nonsteroidal anti-inflammatory drugs; SUA, serum uric acid; Hb, hemoglobin; Alb, albumin.

Missing value: Hb, 419; Alb, 219; prealbumin, 659; sodium, 123; potassium, 141; chloride, 121; phosphate, 135.

### 3.2 Association between SUA and AKI in the development cohort

**FIGURE 1:**
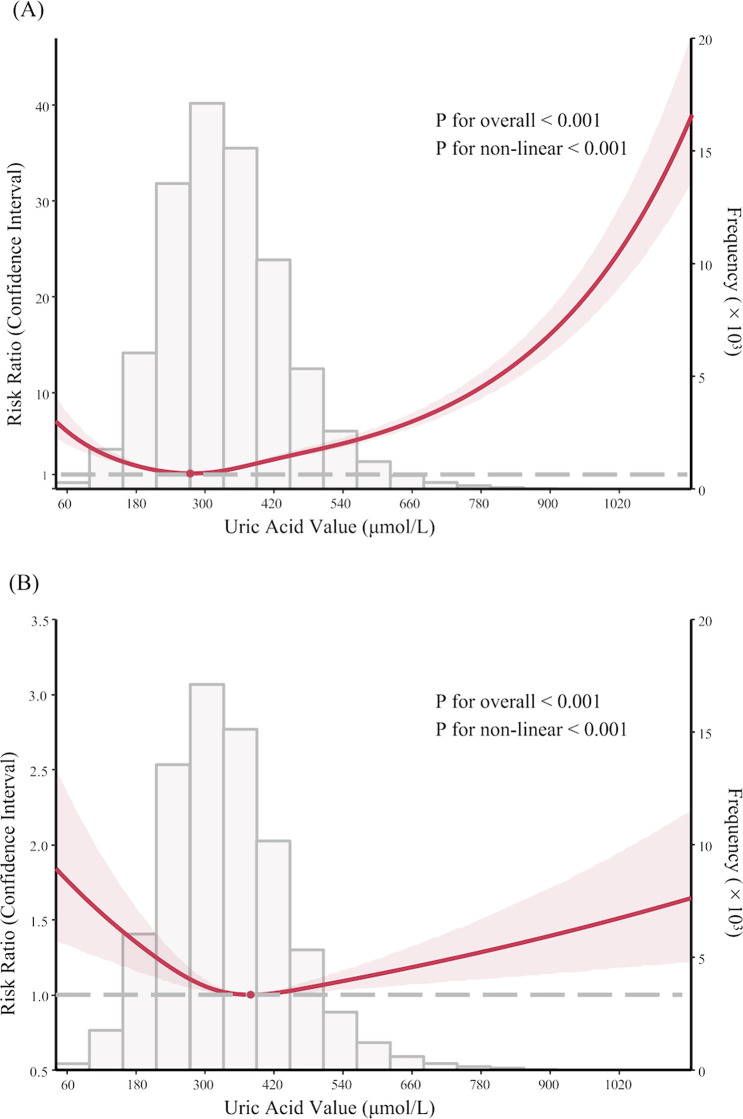
Nonlinear association between serum uric acid levels and acute kidney injury in PKUFH database before and after adjustment, demonstrated by restricted cubic spline analysis. **(A)** Unadjusted Restricted Cubic Spline Plot; **(B)** Fully adjusted Restricted Cubic Spline Plot. **(B)** was adjusted for age, gender, BMI, eGFR at admission, comorbidities (hypertension, diabetes, cardiovascular disease, liver cirrhosis, malignancy), medication (diuretic, NSAIDs, contrast, SUA-lowering medication), ICU admission, laboratory tests (Alb, Hb, prealbumin).

RCS was used to demonstrate the relationship between SUA and the risk of AKI. A nonlinear association revealing a U-shaped risk pattern (P for non-linearity < 0.001) was shown. The slope of the U-shaped curve becomes gentler after adjusting for covariates (Figures 1A, B). Notably, the downward-sloping segment of the curve is steeper than the upward-sloping part, indicating a significantly increased AKI risk associated with decreased SUA.

The incidence of AKI was 4.1% (n = 122), 2.0% (n = 148), 1.9% (n = 529), 2.5% (n = 280), 3.6% (n = 232), and 9.1% (n = 555) for SUA levels of ≤180, 180–240, 240–360, 360–420, 420–480 and >480 μmol/L, respectively. Using patients with SUA levels ranging from 240 to 360 μmol/L as a reference group, the risk of AKI was elevated across all SUA groups except for the 180–240 μmol/L group (RR 1.06, 95% CI 0.88–1.27) in the unadjusted analyses (Table 2, Model 1). Notably, patients with SUA levels ≤180 μmol/L and >480 μmol/L exhibited a 2.17-fold (RR 2.17, 95% CI 1.78–2.64) and a 4.86-fold (RR 4.86, 95% CI 4.32–5.48) increased risk of AKI, respectively. The association between SUA and AKI was not significantly modified by demographic variables but was considerably confounded by eGFR. After adjusting for eGFR, the risk of AKI associated with SUA ≤ 180 μmol/L was significantly increased (RR 2.86, 95% CI 2.35–3.49). Conversely, the risk was notably reduced in the group with SUA > 480 μmol/L (RR 1.39, 95% CI 1.21–1.58). After full adjustment, the associated risk of AKI in SUA ≤ 180 μmol/L (RR 1.92, 95% CI 1.57–2.36) and SUA > 480 μmol/L (RR 1.17, 95% CI 1.03–1.34) was weakened but still demonstrated statistical significance.

**TABLE 2:** Association between staged serum uric acid and acute kidney injury in PKUFH database.

Serum uric acid	No. of events (%)	Risk ratio (95% confidence interval)			
		Model 1	Model 2	Model 3	Model 4
≤180 (N = 3,011)	122 (4.1)	2.17 (1.78, 2.64)	2.05 (1.68, 2.50)	2.86 (2.35, 3.49)	1.92 (1.57, 2.36)
180–240 (N = 7,514)	148 (2.0)	1.06 (0.88, 1.27)	1.04 (0.87, 1.25)	1.35 (1.12, 1.62)	1.12 (0.93, 1.34)
240–360 (N = 28,344)	529 (1.9)	Ref.	Ref.	Ref.	Ref.
360–420 (N = 11,272)	280 (2.5)	1.33 (1.15, 1.54)	1.36 (1.17, 1.57)	0.91 (0.79, 1.06)	0.97 (0.84, 1.13)
420–480 (N = 6,516)	232 (3.6)	1.91 (1.64, 2.23)	2.00 (1.71, 2.34)	1.00 (0.86, 1.17)	1.04 (0.89, 1.22)
>480 (N = 6,118)	555 (9.1)	4.86 (4.32, 5.48)	5.11 (4.52, 5.76)	1.39 (1.21, 1.58)	1.17 (1.03, 1.34)

Model 1: Unadjusted.

Model 2: Adjusted for demographics (age, gender, BMI).

Model 3: Further adjusted for eGFR at admission.

Model 4: Further adjusted for comorbidities (hypertension, diabetes, cardiovascular disease, liver cirrhosis, malignancy), medication (diuretic, NSAIDs, contrast, SUA-lowering medication), ICU admission, laboratory tests (Alb, prealbumin, Hb).

**FIGURE 2:**
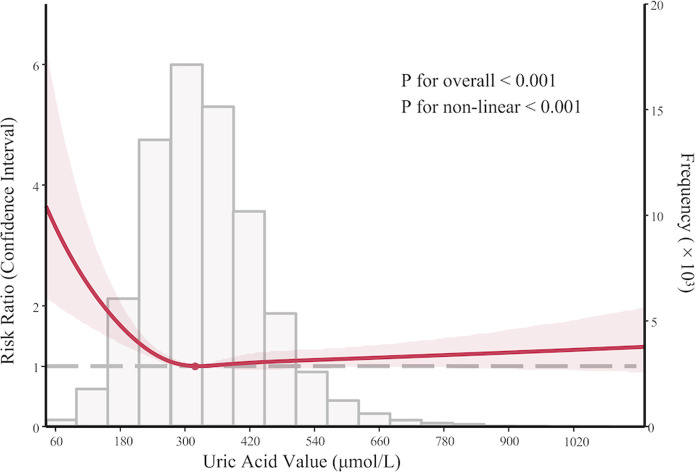
Sensitivity analysis: nonlinear association between serum uric acid levels and acute kidney injury within 7 days after admission in PKUFH database after full adjustment, demonstrated by restricted cubic spline analysis.

After restricting AKI to the 7-day post-admission period, the U-shaped trend in the association between SUA and AKI was also observed, with patients having SUA ≤ 180 μmol/L exhibiting the highest incidence (RR 1.93, 95% CI 1.35–2.76) (Figure 2; Supplementary Table 1). Similar findings were found after excluding patients receiving uric acid-lowering therapy, as those with SUA ≤ 180 μmol/L continued to show the highest risk (RR 1.98, 95% CI 1.61–2.43) (Supplementary Table 2).

### 3.3 Association between SUA and AKI in different eGFR subgroups

**FIGURE 3:**
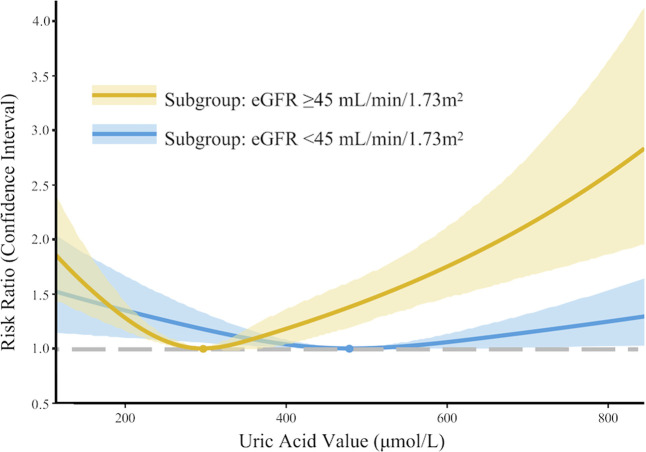
Subgroup analysis: nonlinear association between serum uric acid levels and acute kidney injury in PKUFH after full adjustment, stratified by eGFR (≤45 mL/min/1.73 m^2^ or >45 mL/min/1.73 m^2^).

In the development cohort, when stratified by an eGFR threshold of 45 mL/min/1.73 m^2^, a similar U-shaped risk curve between SUA and AKI was observed (Figure 3); however, the curve was much less steep in the subgroup with eGFR ≤ 45 mL/min/1.73 m^2^. Similar findings were observed in the multivariate Poisson regression analysis. Compared with the reference group (SUA 240–360 μmol/L), the increased AKI risk for SUA≤180 μmol/L (RR 1.40, 95% CI 1.12–1.76) and SUA >480 μmol/L (RR 2.18, 95% CI 1.80–2.64) remains statistically significant only in the subgroup with eGFR > 45 mL/min/1.73 m^2^. While in the subgroup with eGFR ≤ 45 mL/min/1.73 m^2^, the association between SUA and AKI was not significant in all groups (Supplementary Table 3).

### 3.4 Association between SUA and AKI in the external validation cohort

A total of 7,160 patients were enrolled from screened 180,747 patients in the MIMIC-IV database (Supplementary Figure 1B). The median age was 61 (45, 71) years and 49.9% (n = 3,574) was male. The most common comorbidities were malignancy (n = 4,632, 64.7%) and hypertension (n = 2,383, 33.3%). The median SUA was 309.3 (237.9, 398.5) μmol/L and eGFR was 100.2 (78.7, 115.6) mL/min/1.73 m^2^. SUA-lowering medication was prescribed in 29.7% of the entire population. The baseline characteristics of the validation cohort were presented in Supplementary Table 4.

The incidence of AKI was 169 (24.1%), 217 (18.8%), 523 (18.6%), 209 (21.8%), 131 (23.1%), and 287 (29.9%) in groups with SUA ≤ 180, 180–240, 240–360, 360–420, 420–480, and >480 μmol/L, respectively. A similar, though less steep, U-shaped curve between SUA and AKI was observed (Supplementary Figure 2). After full adjustment, the AKI risk remained statistically significant only for SUA ≤ 180 μmol/L (RR 1.22, 95% CI 1.03–1.46). Results of the Poisson regression analysis were displayed in Supplementary Table 5.

## 4 Discussion

In this study, the admission SUA displayed a U-shaped relationship with AKI during hospitalization, with the curve being steeper in the reduced SUA segment than in the elevated SUA segment. This association was confounded by eGFR, particularly for the elevated SUA.

AKI is a serious syndrome in hospitalized patients with no effective treatments available. SUA, functioning as both an antioxidant and a prooxidant, has gained significant attention in the field of AKI. Previous studies have mainly concentrated on the risk of AKI linked to hyperuricemia (Ejaz et al., 2015; Park et al., 2011; Mendi et al., 2017), potentially neglecting the valuable insights that could be gained from analyzing SUA as a continuous measure. Furthermore, many studies were carried out in specific populations, which restricts the generalizability of their findings. To our knowledge, only two studies have evaluated the association between SUA and AKI in general hospitalized patients, and inconsistent findings were observed (Otomo et al., 2016; Cheungpasitporn et al., 2016). Cheungpasitporn et al. (2016) described a positive correlation between SUA level at admission and AKI risk after adjusting for age, gender, BMI, baseline SCr, principal diagnosis comorbidities, and medications. However, selection bias is evident in this study, as only 1.9% (1,435/76,719) of the screened patients had SUA measurements upon admission. The other study in Japan found that both reduced and elevated SUA were associated with a heightened risk of AKI (Otomo et al., 2016), which aligns with our study where a U-shaped risk curve was observed. These findings demonstrate substantial clinical importance in forecasting the risk among hospitalized patients, as reduced SUA levels at admission present a notable risk factor.

Several potential mechanisms may explain the impact of reduced SUA on the increased risk of AKI in our study. First, reduced SUA could result from inadequate intake and is often accompanied by malnutrition, a well-recognized risk factor for AKI (Cao et al., 2021; Beberashvili et al., 2016; Beberashvili et al., 2015). This is demonstrated in our study, where the lowest serum albumin and prealbumin levels were observed in patients with SUA ≤ 180 μmol/L. Second, approximately 90% of filtered uric acid is reabsorbed in the proximal tubule (Bobulescu and Moe, 2012). Reduced SUA may indicate weakened tubular reabsorption disorders due to various conditions such as Fanconi syndrome (Kim and Jun, 2022), which could also increase the vulnerability to AKI (Yeum et al., 2004; Vallon, 2016). Moreover, since SUA contributes to approximately 60% of plasma’s total antioxidant capacity (Yeum et al., 2004), reduced SUA may, therefore, increase the kidney’s vulnerability to oxidative stress, such as ischemia-reperfusion, sepsis, or nephrotoxins (Park et al., 2020). It is important to note that SUA-lowering medication was prescribed to a certain proportion of patients with reduced SUA. Our findings hold significant medical relevance, indicating that treatment intensity must be carefully tailored to prevent excessively low levels of SUA in accordance with guidelines (Richette et al., 2017).

Decreased kidney function can lead to elevated SUA. Conversely, elevated SUA may accelerate kidney impairment due to crystal deposition, inflammation, and oxidative stress (Sánchez-Lozada et al., 2005). Many large observational studies have examined the association between SUA and prognosis in CKD patients, yielding conflicting results (Miyaoka et al., 2014; Russo et al., 2021; Madero et al., 2009). There is no consensus on the targeted SUA range of treatment approaches for CKD patients with asymptomatic hyperuricemia (Eleftheriadis et al., 2017). In a sub-analysis of the Symphony trial involving 1,645 kidney transplant recipients, after adjusting for baseline kidney function, SUA levels no longer showed an independent effect on the progression of kidney dysfunction over the 3 years following transplantation (Meier-Kriesche et al., 2009). We also found that the association between SUA and AKI was modified by baseline kidney function. The increased risk of AKI associated with elevated SUA was only observed in the subgroup with eGFR > 45 mL/min/1.73 m^2^. Similarly, a prospective cohort study involving 3,885 individuals with CKD stages 2–4 revealed that elevated SUA was independently linked to an increased risk of kidney failure in participants with eGFR ≥ 45 mL/min/1.73 m^2^ but not in those with eGFR < 30 mL/min/1.73 m^2^ (Srivastava et al., 2018). Our findings contribute to the evidence that optimal SUA levels require further definition at both the upper and lower thresholds in patients with existing CKD. Additionally, it emphasizes the importance of making appropriate adjustments for kidney function when assessing the effects of SUA.

Our study possesses several noteworthy strengths that contribute to its significance and reliability. First and foremost, it boasts the largest sample size recorded to date for exploring the association between SUA levels and AKI in hospitalized patients. This extensive sample size provides a robust foundation for our analysis and enhances the validity of our conclusions. Second, we conducted meticulous adjustments for a variety of potential confounding factors that could influence the results, such as nutritional indicators and medications known to affect uric acid metabolism. This careful consideration allows us to account for variables that might skew the relationship we are investigating. Lastly, our research included two distinct healthcare centers that serve demographically diverse populations, which significantly enhances the generalizability of our findings. By incorporating this diversity, we ensure that our results are more applicable to a broader range of hospitalized patients, making our study more impactful in the clinical context.

Our study has several limitations. Firstly, pre-admission SCr was unavailable for most patients, which may have led to the underdiagnosis of community-acquired AKI cases. To mitigate this impact, we excluded patients who fulfilled the AKI criteria within 48 h of admission. Secondly, there is a possibility of selection bias due to the retrospective nature, as patients who underwent admission SUA tests may exhibit different clinical characteristics compared to those who did not receive these tests upon admission. A multicenter, prospective study is needed to address this limitation. Lastly, similar to all observational studies, the results of this study may still be affected by unmeasured confounders despite comprehensive adjustments.

## 5 Conclusion

We identified a U-shaped association between admission SUA levels and AKI risk, with both elevated and reduced SUA independently predicting higher AKI incidence in hospitalized patients. This association was significantly influenced by the patients’ baseline kidney function. Additional research is necessary to ascertain the optimal SUA range in hospitalized patients to mitigate the risk of AKI.

## Data Availability

The raw data supporting the conclusion of this article will be made available by the authors, without undue reservation.
