# Supplementary material for: The prognostic value of admission serum uric acid for acute kidney injury: a two-center retrospective analysis
Source: Front Mol Biosci. 2025 Jun 18;12:1635227. doi: 10.3389/fmolb.2025.1635227 (PMC12213346; doi:10.3389/fmolb.2025.1635227)
Supplement: Supplementary file 1 [file Supplementaryfile1.docx]

Supplementary Material

# Supplementary Figures and Tables

## Supplementary Figures

##
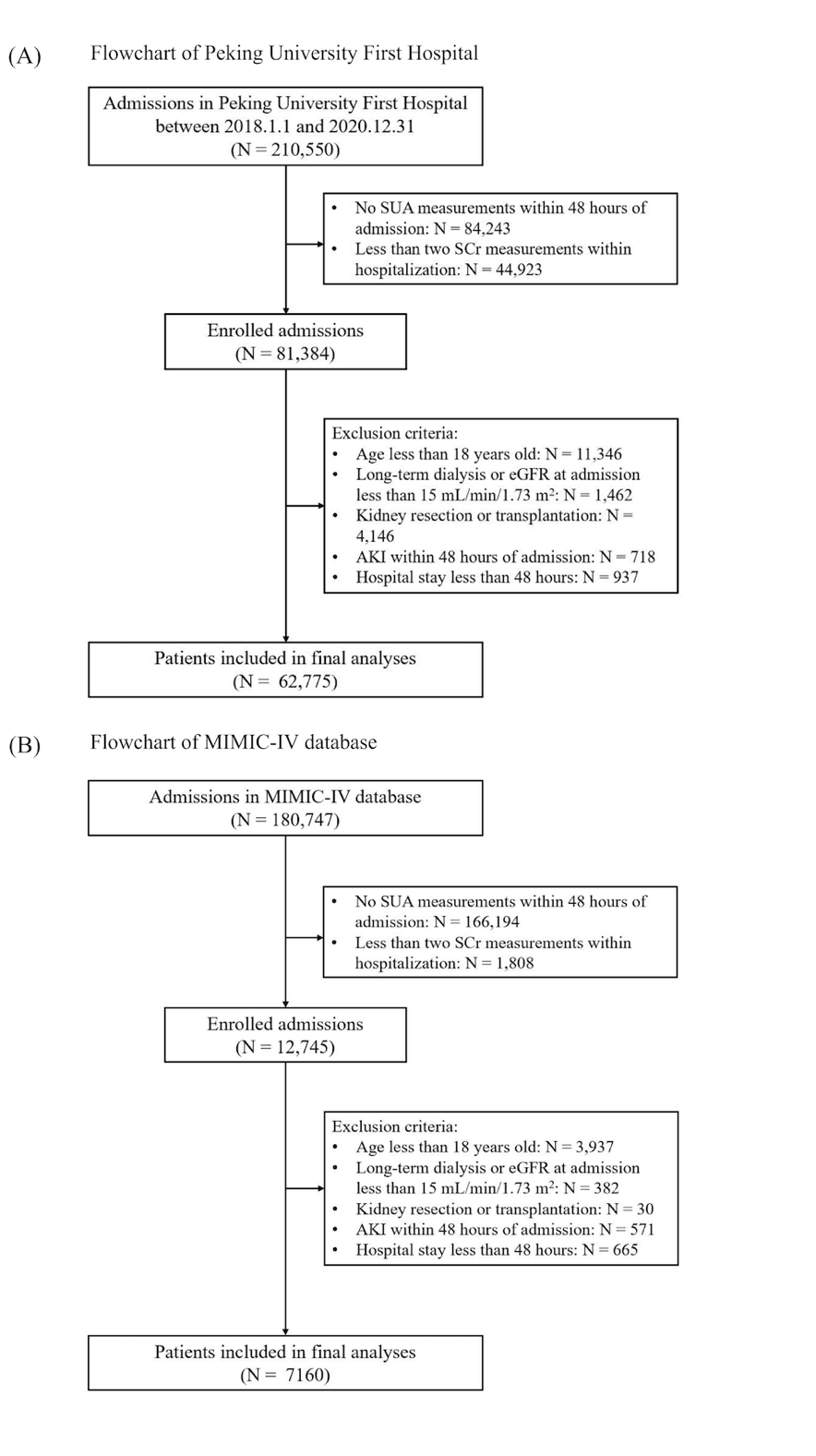


**Supplementary Figure 1.** Flowchart.


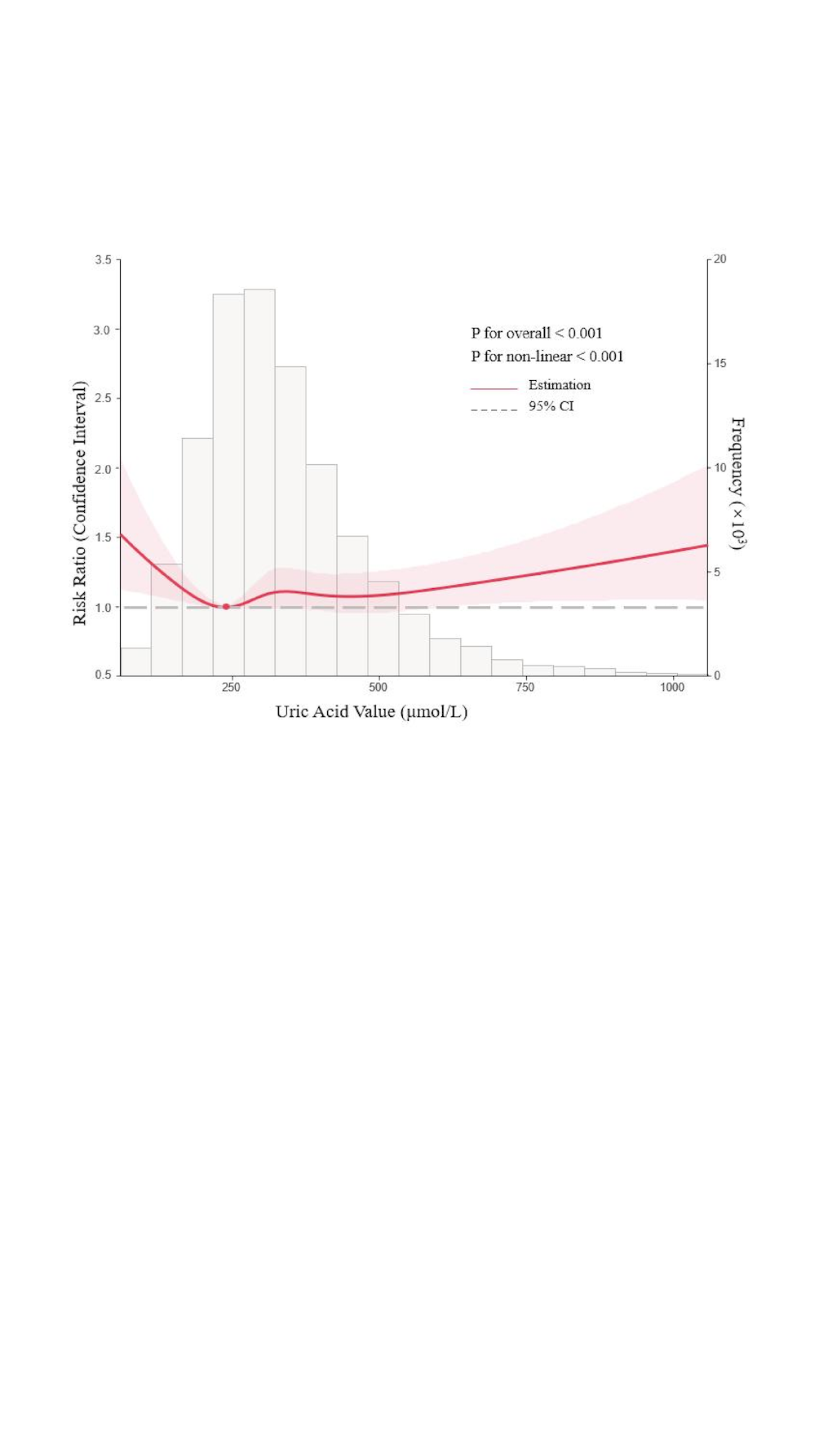


**Supplementary Figure 2.** Nonlinear association between serum uric acid levels and acute kidney injury in MIMIC-IV database after adjustment, demonstrated by restricted cubic spline analysis.

## Supplementary Tables

**Supplementary Table 1. Association between serum uric acid and acute kidney injury within 7 days after admission in PKUFH database.**

|  | **No. of Events (%)** | **Risk Ratio (95 Confidence Interval)** | | | |
| --- | --- | --- | --- | --- | --- |
|  |  | Model 1 | Model 2 | Model 3 | Model 4 |
| ≤ 180 (N = 3011) | 37(1.2) | 1.72(1.21,2.44) | 1.63(1.14,2.32) | 2.38(1.67,3.38) | 1.93(1.35,2.76) |
| 180-240 (N = 7514) | 62(0.8) | 1.15(0.87,1.53) | 1.14(0.86,1.52) | 1.56(1.17,2.07) | 1.36(1.02,1.81) |
| 240-360 (N = 28344) | 203(0.7) | Ref. | Ref. | Ref. | Ref. |
| 360-420 (N = 11272) | 115(1.0) | 1.42(1.13,1.79) | 1.45(1.15,1.83) | 0.87(0.69,1.10) | 0.92(0.73,1.16) |
| 420-480 (N = 6516) | 107(1.6) | 2.29(1.81,2.90) | 2.41(1.90,3.05) | 1.01(0.80,1.28) | 1.06(0.83,1.34) |
| >480 (N=6118) | 269(4.4) | 6.14(5.12,7.37) | 6.44(5.35,7.75) | 1.29(1.06,1.58) | 1.13(0.93,1.38) |

Model 1: Unadjusted.

Model 2: Adjusted for age, gender, BMI.

Model 3: further adjusted for eGFR at admission

Model 4: further adjusted for comorbidities (hypertension, diabetes, cardiovascular disease, liver cirrhosis, malignancy), medication (diuretic, NSAIDs, contrast, UA-lowering medication), ICU admission, laboratory tests (Alb, Hb, prealbumin)

**Supplementary Table 2. Association between serum urine acid and acute kidney injury after excluding** **patients who were treated with serum uric acid-lowering medications in PKUFH database.**

|  | **No. of Events (%)** | **Risk Ratio (95 Confidence Interval)** | | | |
| --- | --- | --- | --- | --- | --- |
|  |  | Model 1 | Model 2 | Model 3 | Model 4 |
| ≤ 180 (N = 2975) | 120(4.0) | 2.21(1.81,2.69) | 2.07(1.69,2.52) | 2.91(2.38,3.55) | 1.98(1.61,2.43) |
| 180-240 (N = 7431) | 141(1.9) | 1.04(0.86,1.25) | 1.02(0.85,1.23) | 1.34(1.11,1.61) | 1.11(0.92,1.34) |
| 240-360 (N = 27938) | 511(1.8) | Ref. | Ref. | Ref. | Ref. |
| 360-420 (N = 11055) | 271(2.5) | 1.34(1.16,1.55) | 1.37(1.18,1.59) | 0.91(0.79,1.06) | 0.98(0.84,1.14) |
| 420-480 (N = 6319) | 225(3.6) | 1.95(1.66,2.28) | 2.05(1.75,2.41) | 1.00(0.85,1.18) | 1.05(0.89,1.23) |
| >480 (N = 5749) | 515(9.0) | 4.90(4.33,5.54) | 5.16(4.56,5.85) | 1.35(1.18,1.55) | 1.15(1.00,1.31) |

Model 1: Unadjusted.

Model 2: Adjusted for age, gender, BMI.

Model 3: further adjusted for eGFR at admission

Model 4: further adjusted for comorbidities (hypertension, diabetes, cardiovascular disease, liver cirrhosis, malignancy), medication (diuretic, NSAIDs, contrast), ICU admission, laboratory tests (Alb, Hb, prealbumin)

**Supplementary Table 3. Subgroup analysis stratified by eGFR in PKUFH database.**

| **Subgroup** | **No. of Events (%)** | **Risk Ratio (95 Confidence Interval)** | |
| --- | --- | --- | --- |
|  |  | Unadjusted | Adjusted |
| **eGFR>45 mL/min/1.73 m^2^** |  |  |  |
| ≤ 180 (N = 2932) | 106(3.6) | 2.83(2.28,3.52) | 1.40(1.12,1.76) |
| 180-240 (N = 7371) | 112(1.5) | 1.19(0.96,1.47) | 0.99(0.80,1.23) |
| 240-360 (N = 27476) | 351(1.3) | Ref. | Ref. |
| 360-420 (N = 10439) | 164(1.6) | 1.23(1.02,1.48) | 1.25(1.04,1.51) |
| 420-480 (N = 5717) | 121(2.1) | 1.66(1.35,2.04) | 1.57(1.27,1.94) |
| >480 (N = 4206) | 167(4.0) | 3.11(2.59,3.74) | 2.18(1.80,2.64) |
| **eGFR≤45 mL/min/1.73 m^2^** |  |  |  |
| ≤ 180 (N = 79) | 16(20.3) | 0.99(0.59,1.65) | 1.19(0.71,2.00) |
| 180-240 (N = 143) | 36(25.2) | 1.23(0.86,1.76) | 1.05(0.73,1.51) |
| 240-360 (N = 868) | 178(20.5) | Ref. | Ref. |
| 360-420 (N = 833) | 116(13.9) | 0.68(0.54,0.86) | 0.78(0.62,0.99) |
| 420-480 (N = 799) | 111(13.9) | 0.68(0.53,0.86) | 0.75(0.59,0.95) |
| >480 (N = 1912) | 388(20.3) | 0.99(0.83,1.18) | 0.98(0.82,1.17) |

Adjusted for age, gender, BMI, comorbidities (hypertension, diabetes, cardiovascular disease, liver cirrhosis, malignancy), medication (diuretic, NSAIDs, contrast, SUA-lowering medication), ICU admission, laboratory tests (Alb, Hb, prealbumin)

**Supplementary Table 4. Baseline characteristics of MIMIC-IV database.**

|  | **Total**  **(N = 7160)** | **< 180**  **(N =** 702**)** | **180-240**  **(N =** 1156**)** | **240-360**  **(N =** 2816**)** | **360-420**  **(N =** 959**)** | **420-480**  **(N =** 568**)** | **> 480**  **(N = 959)** |
| --- | --- | --- | --- | --- | --- | --- | --- |
| Age (years) | 61(45,71) | 60(47.2,70) | 58(42,69) | 59(40,70) | 62(45,72) | 64(48,73) | 67(57,78) |
| Male | 3574(49.9) | 248(35.3) | 490(42.4) | 1377(48.9) | 545(56.8) | 323(56.9) | 591(61.6) |
| White | 4937(69.0) | 494(70.4) | 788(68.2) | 1952(69.3) | 652(68.0) | 407(71.7) | 644(67.2) |
| BMI (kg/m^2^) | 27.8(23.9,32.4) | 27.5(23.1,32.6) | 27.7(23.7,32.0) | 27.8(24.0,32.4) | 28.0(24.3,32.4) | 27.9(23.8,32.6) | 28.2(24.3,32.8) |
| Baseline eGFR (mL/min/1.73m^2^) | 100.2(78.7,115.6) | 108.1(97.1,120.5) | 107.3(95.2,121.3) | 103.5(89.6,118.7) | 95.9(73.3,111.8) | 87.3(61.7,104.2) | 60.9(40.1,88.9) |
| Baseline SCr (μmol/L) | 0.7(0.6,1.0) | 0.6(0.4,0.7) | 0.6(0.5,0.8) | 0.7(0.5,0.9) | 0.8(0.6,1.0) | 0.9(0.7,1.1) | 1.2(0.9,1.6) |
| ICU admission | 725(10.1) | 89(12.7) | 101(8.7) | 214(7.6) | 76(7.9) | 59(10.4) | 186(19.4) |
| **Comorbidities, n(%)** |  |  |  |  |  |  |  |
| Hypertension | 2383(33.3) | 225(32.1) | 373(32.3) | 950(33.7) | 342(35.7) | 213(37.5) | 280(29.2) |
| Diabetes | 1325(18.5) | 96(13.7) | 169(14.6) | 438(15.6) | 194(20.2) | 130(22.9) | 298(31.1) |
| Cardiovascular disease | 1318(18.4) | 78(11.1) | 117(10.1) | 421(15.0) | 168(17.5) | 148(26.1) | 386(40.3) |
| Liver cirrhosis | 133(1.9) | 13(1.9) | 20(1.7) | 34(1.2) | 15(1.6) | 16(2.8) | 35(3.6) |
| Malignancy | 4632(64.7) | 499(71.1) | 793(68.6) | 1850(65.7) | 606(63.2) | 350(61.6) | 534(55.7) |
| **Medication, n(%)** |  |  |  |  |  |  |  |
| Diuretic | 1386(19.4) | 70(10.0) | 143(12.4) | 418(14.8) | 208(21.7) | 154(27.1) | 393(41.0) |
| NSAIDs | 5152(72.0) | 502(71.5) | 799(69.1) | 2018(71.7) | 700(73.0) | 403(71.0) | 730(76.1) |
| Contrast | 250(3.5) | 23(3.3) | 35(3.0) | 98(3.5) | 38(4.0) | 22(3.9) | 34(3.5) |
| SUA-lowering medication | 2123(29.7) | 211(30.1) | 276(23.9) | 684(24.3) | 277(28.9) | 240(42.3) | 435(45.4) |
| **Lab testing** |  |  |  |  |  |  |  |
| Hb (g/dL) | 10.1(8.6,11.7) | 9.5(8.2,11.1) | 10.0(8.6,11.4) | 10.3(8.8,11.9) | 10.3(8.9,11.9) | 10.1(8.4,11.8) | 10.0(8.4,11.6) |
| Alb (g/dL) | 3.7(3.2,4.0) | 3.6(3.1,3.9) | 3.7(3.3,4.0) | 3.7(3.3,4.1) | 3.7(3.3,4.1) | 3.7(3.2,4.0) | 3.5(3.1,3.9) |

body mass index, BMI; estimated glomerular filtration rate, eGFR; serum creatinine, SCr; intensive care unit, ICU; nonsteroidal anti-inflammatory drugs, NSAIDs; serum uric acid, SUA; hemoglobin, Hb; albumin, Alb

Missing value: BMI, 3504; Hb, 32; Alb, 2289.

**Supplementary Table 5. Association between serum urine acid and acute kidney injury in MIMIC-IV database.**

|  | **No. of Events (%)** | **Risk Ratio (95 Confidence Interval)** | | | |
| --- | --- | --- | --- | --- | --- |
|  |  | Model 1 | Model 2 | Model 3 | Model 4 |
| ≤ 180 (N = 702) | 169(24.1) | 1.30(1.09,1.54) | 1.29(1.08,1.53) | 1.35(1.13,1.61) | 1.22(1.03,1.46) |
| 180-240 (N = 1156) | 217(18.8) | 1.01(0.86,1.19) | 1.02(0.87,1.19) | 1.05(0.90,1.23) | 1.04(0.88,1.22) |
| 240-360 (N = 2816) | 523(18.6) | Ref. | Ref. | Ref. | Ref. |
| 360-420 (N = 959) | 209(21.8) | 1.17(1.00,1.38) | 1.13(0.97,1.33) | 1.09(0.93,1.23) | 1.10(0.93,1.29) |
| 420-480 (N = 568) | 131(23.1) | 1.24(1.03,1.50) | 1.18(0.98,1.43) | 1.09(0.90,1.33) | 1.00(0.82,1.22) |
| >480 (N= 959) | 287(29.9) | 1.61(1.40,1.86) | 1.46(1.26,1.69) | 1.25(1.06,1.47) | 1.09(0.92,1.28) |

Model 1: Unadjusted.

Model 2: Adjusted for age, gender, BMI.

Model 3: further adjusted for eGFR at admission

Model 4: further adjusted for comorbidities (hypertension, diabetes, cardiovascular disease, liver cirrhosis, malignancy), medication (diuretic, NSAIDs, contrast, SUA-lowering medication), ICU admission, laboratory tests (Alb, Hb)
